# The Poly-Arginine Peptide R18D Inhibits Amyloid-Beta (Aβ) Aggregation and Aβ-Induced Cytotoxicity, Reduces Intracellular Tau Aggregation, and Exhibits Oral Bioavailability

**DOI:** 10.3390/biomedicines14071564

**Published:** 2026-07-13

**Authors:** Vaishali Bagda, Zainab H. Farooz, Neville W. Knuckey, Samantha M. South, Stuart K. Gribble, Maitri Tomar, Prashant Bharadwaj, Ajish Ariyath, Kevin Taddei, Ralph N. Martins, Bruno P. Meloni

**Affiliations:** 1 Perron Institute for Neurological and Translational Science, Nedlands, WA 6009, Australia; vaishali.bagda@perron.uwa.edu.au (V.B.);; 2 School of Human Sciences, The University of Western Australia, Nedlands, WA 6009, Australia; 3 Centre for Neuromuscular and Neurological Disorders, The University of Western Australia, Nedlands, WA 6009, Australia; 4 Department of Neurosurgery, Sir Charles Gairdner Hospital, QEII Medical Centre, Nedlands, WA 6009, Australia; 5 Argenica Therapeutics, Nedlands, WA 6009, Australia; 6 School of Biological Sciences, The University of Western Australia, Crawley, WA 6009, Australia; 7 Centre of Excellence for Alzheimer’s Disease Research and Care, School of Medical and Health Sciences, Edith Cowan University, Joondalup, WA 6027, Australia; 8 Alzheimer’s Research Australia, Ralph and Patricia Sarich Neuroscience Research Institute, Nedlands, WA 6009, Australia; 9 Medical School, The University of Western Australia, Nedlands, WA 6009, Australia; 10 Department of Biomedical Sciences, Macquarie University, Sydney, NSW 2109, Australia

**Keywords:** Alzheimer’s disease, amyloid-beta, tau protein, cationic arginine-rich peptides, R18D, SH-SY5Y cells, primary cortical neurons, MC65 cells, tau seeds

## Abstract

**Background/Objectives**: Effective disease-modifying therapies targeting pathogenic proteins associated with Alzheimer’s disease (AD) remain limited. This study investigated the therapeutic potential of the neuroprotective, cationic arginine-rich peptide R18D to mitigate the pathogenic effects of amyloid-beta (Aβ) and tau associated with AD. **Methods**: R18D was examined for its ability to inhibit Aβ aggregation in a cell-free assay, attenuate Aβ-induced cytotoxicity in MC65 cells, and suppress intracellular tau aggregation in two neural cell models. Intracellular tau aggregation was quantified using a homogeneous time-resolved fluorescence assay. Additionally, a pilot pharmacokinetic study of R18D was conducted in mice following oral gavage administration. **Results:** In the cell-free assay, R18D inhibited Aβ aggregation by up to 65%. In human MC65 cells induced to overexpress APP-C99 and accumulate Aβ, treatment with R18D inhibited cellular toxicity by as much as 100%. Preformed tau seeds were applied to human SH-SY5Y cells and rat primary cortical neurons to induce intracellular tau aggregation, and tau levels were quantified after 48 h. Exposure to tau seeds induced robust tau aggregation in both cellular models, which was significantly attenuated by R18D treatment, reducing aggregation by 34.8% in SH-SY5Y cells and 49.9% in cortical neurons. Pharmacokinetic studies demonstrated that R18D was detectable in plasma at 30 and 60 min following oral administration in mice. **Conclusions**: Together, these results demonstrate that R18D can modulate both Aβ and tau pathologies in vitro and is orally bioavailable, supporting its further evaluation as a therapeutic candidate for AD and other tau-associated neurodegenerative disorders.

## 1. Introduction

Alzheimer’s disease (AD) is the most prevalent form of dementia, comprising 60–80% of cases. It is estimated that AD and other dementias affect over 50 million people worldwide, a figure projected to rise to around 131 million by 2050 [1]. AD and other dementias are characterized by progressive cognitive decline, with substantial societal and economic impact, including annual healthcare costs estimated to be over US $1 trillion globally [2]. Despite significant research advances, disease-modifying therapies remain limited, and currently approved drugs provide only symptomatic relief or modest benefit in terms of slowing disease progression [3,4,5]. Indeed, a recent Cochrane review of different monoclonal antibodies developed to clear amyloid deposits in patients with AD found that their clinical effects were “either absent or consistently small” [6].

AD pathology is defined by the accumulation and aggregation of amyloid-beta (Aβ) in extracellular plaques, and intracellular neurofibrillary tangles (NFTs) composed of hyperphosphorylated aggregated tau protein [7]. Aβ, produced by sequential proteolytic cleavage of the amyloid precursor protein (APP), aggregates into oligomers, protofibrils, and fibrils, with Aβ_1–42_ species being particularly prone to aggregation and neurotoxicity. While initially considered inert deposits, Aβ aggregates, especially soluble oligomers, disrupt synaptic signaling, impair calcium homeostasis, and trigger neuroinflammatory and apoptotic cell death pathways [8,9]. These findings highlight the necessity of developing therapeutics that mitigate Aβ toxicity and aggregation, while restoring intracellular proteostasis.

In vitro, Aβ aggregation can be modeled using cell-free thioflavin-T assays, which allow precise monitoring of fibril formation and evaluation of anti-aggregation compounds [10]. To further investigate intracellular Aβ toxicity, the human neuroblastoma MC65 cell line provides a well-characterized model [11,12,13]. These cells express the APP-C99 fragment upon tetracycline withdrawal, resulting in intracellular Aβ accumulation and cell death.

Parallel to Aβ pathology, tau is a microtubule-associated protein predominantly expressed in hippocampal and cortical neurons, where it plays vital roles in maintaining cytoskeletal integrity, axonal transport and synaptic plasticity [7,14]. Under pathological conditions such as AD, frontotemporal dementia, and other tauopathies, tau undergoes hyperphosphorylation, leading to its dissociation from microtubules and aggregation into oligomers, paired helical filaments, and NFTs [14]. According to the “prion-like hypothesis”, misfolded tau aggregates propagate between neurons via trans-synaptic transfer or endocytic uptake, where they act as seeds that induce further tau misfolding, thereby driving the progressive spread of pathology in AD [15,16,17,18]. In vitro models using SH-SY5Y neuroblastoma cell lines and primary cortical neurons have become key platforms for investigating tau aggregation and evaluating anti-aggregation compounds, offering advantages over in vivo systems in scalability, experimental throughput, and ethical feasibility [19,20].

Given the limited efficacy of current AD therapies, there is growing interest in multimodal strategies that target core pathological mechanisms. Neuroprotective cationic arginine-rich peptides (CARPs) are emerging as promising therapeutic candidates due to their ability to cross cell membranes and the blood–brain barrier, antagonize calcium influx, preserve mitochondrial integrity, inhibit protein aggregation and suppress neuroinflammatory responses [21]. R18D, a proteolytically stable D-enantiomer poly-arginine peptide [22,23], along with its L-enantiomer (R18), has demonstrated consistent neuroprotective efficacy across multiple experimental models, including stroke [22,24,25], excitotoxicity [23,26,27], hypoxic–ischemic encephalopathy [27] and traumatic brain injury [28].

In this study, we utilized multiple in vitro models to investigate the effects of R18D on key pathological aspects of AD, including a cell-free Aβ aggregation assay, an intracellular Aβ toxicity model using human MC65 cells, and intracellular tau aggregation models in human SH-SY5Y cells and rat primary cortical neurons. These models were used to evaluate the therapeutic potential of R18D in inhibiting Aβ aggregation, reducing Aβ-induced cytotoxicity, and suppressing intracellular tau aggregation. Additionally, R18D was evaluated for oral bioavailability, as oral delivery represents a highly favorable administration route for the long-term treatment of AD.

## 2. Materials and Methods

### 2.1. R18D Peptide

R18D (H-rrrrrrrrrrrrrrrrrr-NH2; r = D-arginine) was synthesized by AmbioPharm (North Augusta, SC, USA) and purified by high-performance liquid chromatography to a purity of 99%. Peptide content was determined to be 51% based on hydrolysis and amino acid liquid chromatography analysis. For cell culture studies, a 500 µM stock solution of R18D was initially prepared in Baxter water and stored at 4 °C. Working solutions of R18D were prepared in DMEM/2% FBS or NB/B27 culture media for SH-SY5Y cell and cortical neuronal studies, respectively. For the oral pharmacokinetic study, lyophilized R18D was weighed and reconstituted in saline (0.9% sodium chloride for injection; Baxter, Deerfield, IL, USA) at a concentration of 283 mg/mL.

### 2.2. Cell-Free Aβ_1–42_ Aggregation Assay

Human recombinant monomeric Aβ_1–42_ (Aβ_1–42_; rPeptide Cat#: A-1167-2) was prepared as a 222 µM stock in 5 mM Tris buffer and diluted in Tris-buffer saline (TBS; 50 mM Tris, 150 mM NaCl, pH 7.4) to a 2× Aβ (50 µM) working solution. The Thioflavin T (ThT) aggregation assay was performed in black microtiter plates, with each well containing 20 µL of 2× Aβ, 10 µL ThT (160 µM), and 10 µL of 4× R18D stock (10, 30, or 100 µM), resulting in final concentrations of 25 µM Aβ, 40 µM ThT, and 2.5, 7.5 or 25 µM R18D. Fluorescence measurements were recorded using a Cytation 5 multimode reader (BioTek, Winooski, VT, USA) at 450 nm excitation and 485 nm emission. A baseline reading was taken immediately after adding test components to wells, followed by kinetic measurements every 10 min for 16 h at 37 °C.

### 2.3. MC65 Cells and Intracellular Aβ_1–42_ Cytotoxicity Model

**Figure 1:**
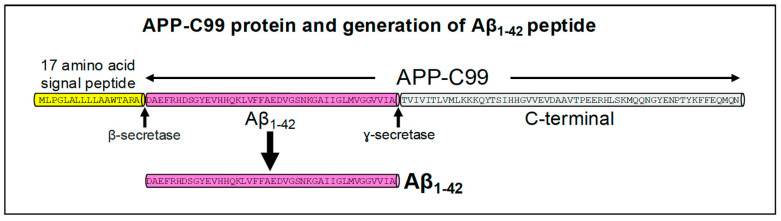
Schematic illustrating the proteolytic processing of APP-C99 by β- and γ-secretases, resulting in the generation of Aβ.

MC65 cells are a genetically modified human neuroblastoma cell line (parent cell line SK-N-MC) that accumulate intracellular Aβ through conditional expression of the carboxy-terminal 99 residues of amyloid-β precursor protein (APP-C99; Figure 1) under tetracycline regulation [11]. Upon removal of tetracycline from the culture medium, APP-C99 is cleaved by endogenous β- and γ-secretases, leading to the generation of Aβ (Figure 1).

The MC65 cells were cultured in Dulbecco’s modified eagle medium DMEM (Thermo Fisher Scientific, Waltham, MA, USA), supplemented with 10% fetal bovine serum (FBS) and penicillin (0.12 mg/mL), streptomycin (0.2 mg/mL), and tetracycline (3 µg/mL; Sigma-Aldrich, St. Louis, MO, USA; T8032) in a CO_2_ incubator (5% CO_2_, 95% air balance, 93% humidity) at 37 °C. Cells were grown in a 25 cm^2^ flask and passaged by trypsinization (TrypLETM Express, Thermo Fisher Scientific) every 2–3 days when the cell monolayer reached approximately 80–90% confluency. To maintain consistency, only cells with fewer than 20 passages relative to the originally characterized cell line were used. Twenty-four hours after plating, G418 (0.2 mg/mL; Sigma-Aldrich; G5013) was added to the culture media for selection of genetically modified cells.

For studies using 96-well plates, cells were seeded at a density of 50,000 cells/well in 100 µL of DMEM/10% FBS and tetracycline. After 48 h of incubation, the medium was replaced with DMEM/10% FBS with (normal control), without tetracycline to induce Aβ production (Aβ control) or without tetracycline plus R18D at final concentrations of 0.03125, 0.0625, 0.125, 0.25 or 0.5 µM (Aβ/R18D control).

For characterization of the Aβ toxicity model, cells were incubated for 1 to 4 days following tetracycline removal, whereas R18D treatment studies were conducted for 72 h post-tetracycline removal.

Cell viability and cell death were assessed using MTS (Promega, Madison, WI, USA; #G3582) and lactate dehydrogenase (LDH) (Promega; #G1780) assays, respectively. The MTS assay measures viability based on the reduction of MTS to a formazan product by metabolically active cells. The MTS substrate is added to wells and after a 1–2 h incubation in the CO_2_ incubator, absorbance is measured at 490 nm. MTS absorbance data were presented graphically or normalized to the untreated control, which was defined as 100% cell viability, and presented graphically as percentage cell viability. The LDH assay quantifies cell death by measuring LDH release from dead cells, which is detected biochemically. An equal volume of the LDH reaction mix is added to the cell culture supernatant removed from the wells. After 20 min incubation at 37 °C, absorbance is measured at 490 nm and presented graphically to represent cell death.

### 2.4. SH-SY5Y Cell Cultures

SH-SY5Y human neuroblastoma cells were cultured in DMEM, supplemented with 2% or 10% FBS, penicillin (0.12 mg/mL) and streptomycin (0.2 mg/mL). Cells were maintained in 25 cm^2^ culture flasks in a CO_2_ incubator and passaged every 2–3 days by trypsinization (TrypLETM Express, Grand Island, NY, USA) or when the monolayer reached approximately 90% confluency. For studies using 96-well plates, approximately 45,000 cells per well were seeded in 50 µL of DMEM/2% FBS and used 24 h after plating.

### 2.5. Primary Cortical Neuronal Cultures

Animal procedures were approved by the University of Western Australia Animal Ethics Committee (Approval Number: 2020/ET000227) and conducted in accordance with the Animal Welfare Act 2002 (Western Australia) and the Australian Code for the Care and Use of Animals for Scientific Purposes (8th edition, 2013).

Rat primary cortical neuronal cultures were established using brain tissue isolated from embryonic day 18 pups, as previously described [29]. Dissociated cortical neurons were seeded into poly-D-lysine-coated wells of 96-well plates (~52,000 neurons per well) in Neurobasal (NB; Thermo Fisher Scientific) medium supplemented with 2% B27 (Thermo Fisher Scientific) and containing penicillin (0.12 mg/mL) and streptomycin (0.2 mg/mL), in a final volume of 120 µL. On day in vitro 4, 50 µL of fresh NB/B27 medium was added to each well. On day in vitro 8, 70 µL of media was removed and replaced with 70 µL fresh NB/B27. Plates were maintained in a CO_2_ incubator, and neuronal cultures were used for experiments 10–12 days after plating. Based on previous studies from our laboratory [29], these cultures typically consist of >98% neurons, with astrocytes comprising the majority of the remaining non-neuronal cells.

### 2.6. Tau Protein Aggregates/Seeds

Recombinant human tau protein (mutated P301S) aggregates/seeds (Note: terms tau aggregates and tau seeds are used interchangeably) used for in vitro cell culture studies were obtained from Abcam (Cambridge, UK, Cat#: ab246003). Prior to use, aliquots containing tau aggregates (15 µL in 0.5 mL microtubes) were placed in a sonicating water bath (Waterbath Branson 3210, Branson Ultrasonics, Danbury, CT, USA) for 2 min to ensure uniform dispersion of aggregates. The dispersed tau aggregates were used to prepare working concentrations of the protein used for all cell culture studies.

### 2.7. Assessment of R18D Toxicity in SH-SY5Y and Cortical Neuronal Cell Cultures

To determine a suitable concentration range of R18D for subsequent intracellular tau aggregation studies, peptide toxicity was first assessed in SH-SY5Y cell and primary cortical neuronal cultures. Cells were treated with R18D at concentrations of 0.015, 0.03, 0.06, 0.12, 0.25, 0.5, 1, 2 or 4 µM for 48 h. Cell viability and cell death were assessed using MTS and LDH assays, respectively (see Section 2.3 for details).

### 2.8. Intracellular Tau Aggregation Models

SH-SY5Y cells and primary cortical neurons were used to develop intracellular tau aggregation models. To establish optimal tau seed concentrations for inducing aggregation, dose–response experiments were performed on both cell types. Intracellular tau aggregation was quantified using a homogenous time-resolved fluorescence (HTRF) assay, which specifically detects aggregated tau protein (see Section 2.10 for details).

SH-SY5Y cell model: Tau seeds (0.025, 0.05, 0.1, 0.2 or 0.4 µg per well) were prepared in 50 µL of DMEM/2% FBS and added to wells containing 50 µL of medium in a 96-well plate, resulting in monomeric tau concentrations of 0.005, 0.01, 0.02, 0.04, 0.08 and 0.16 µM (tau seeds), respectively. Cells were incubated with tau seeds for 2 h to allow cellular uptake. After the 2 h incubation, 50 µL of DMEM/2% FBS was added to each well to replicate conditions for subsequent compound testing (i.e., R18D), followed by a further 46 h incubation, resulting in a total tau seed exposure time of 48 h.

Cortical neuronal model: Medium was removed from wells (96-well plate) and tau seeds (0.025, 0.05, 0.1, 0.2, or 0.4 µg per well) prepared in 100 µL NB/B27 were added, resulting in monomeric tau concentrations of 0.005, 0.01, 0.02, 0.04, 0.08 and 0.16 µM (tau seeds), respectively. After a 2 h incubation, an additional 50 µL of NB/B27 was added, followed by a further 46 h incubation, resulting in a total tau seed exposure time of 48 h.

### 2.9. Assessment of R18D in Inhibiting Intracellular Tau Aggregation

The SH-SY5Y cells and cortical neurons were used to examine the inhibitory effect of R18D on intracellular tau aggregation.

SH-SY5Y cell model: Tau seeds were added to culture wells at a concentration of 0.08 µM and incubated for 2 h. Subsequently, 50 µL of DMEM/2% FBS containing R18D at 3X the target concentration was added, resulting in final peptide concentrations of 0.025, 0.05, 0.1, 0.2 and 0.4 µM, followed by a further 46 h incubation. Controls consisted of SH-SY5Y cells treated with DMEM/2% FBS either with or without tau seeds.

Cortical neuronal model: Tau seeds were added to culture wells at a concentration of 0.08 µM for 2 h. Subsequently, 50 µL of NB/B27 containing R18D at 3X the target concentration was added, resulting in final peptide concentrations of 0.0625, 0.125, 0.25 and 0.5 µM, followed by a further 46 h incubation. Controls consisted of neuronal culture wells treated with NB/B27 with and without tau seeds.

### 2.10. Tau Aggregate Homogenous Time-Resolved Fluorescence (HTRF) Assay

Intracellular tau aggregates were quantified using the HTRF tau aggregate kit (Revvity, Waltham, MA, USA, Cat#: 6FTAUPEG) as per the manufacturer’s instructions. Briefly, cells were lysed with 50 µL of lysis buffer per well and plates shaken at 300 rpm for 20 min at room temperature. Subsequently, 7.5 µL of cell lysate was transferred to a 384-well plate and incubated with 3.75 µL each of tau aggregate-specific acceptor and donor antibodies for 2 h at room temperature with shaking (300 rpm). Fluorescence was measured using a CLARIOstar plate reader, using filters for simultaneous detection of donor (620 nm) and acceptor (665 nm) emissions following excitation at 337 nm. Intracellular tau aggregate levels were calculated as the ratio of acceptor to donor emissions (665 nm/620 nm × 104 = HTRF ratio) as stated in the manual.

### 2.11. Pharmacokinetic Study of R18D Following Oral Administration in Mice

Given that oral dosing is a convenient route for long-term treatment of AD, we performed a proof-of-concept pharmacokinetic study on mice to assess the oral bioavailability of R18D. The study was approved by the University of Western Australia Animal Ethics Committee (Approval Number: 2023/ET000282) and followed guidelines described above (See Section 2.5). Male C57Bl6/J mice (5–6 weeks of age) were obtained from OzGene ARC (Perth, Australia). Animals were group-housed (2–5 animals per cage) under a 12 h day/night cycle (7 am to 7 pm) with ad libitum access to food and water.

Mice received a single dose of R18D (20,000 nmol/kg in 0.2 mL saline) via oral gavage. The dose was selected based a 20-fold higher maximum intravenous dose of R18D (1000 nmol/kg) previously used in a rat study [24], together with data from oral dosing studies of other CARPs in mice [30,31,32]. Animals (n = 3 per time point) were euthanized at 30, 60 and 120 min post-administration. Mice assigned to each post-R18D administration blood collection time point were dosed sequentially on the same day. Euthanasia was performed by intraperitoneal injection of an overdose of sodium pentobarbitone (0.1 mL; Lethabarb, Virbac, Carros, France). Blood (0.5–0.8 mL) was collected via cardiac puncture into K2-EDTA tubes (BD Vacutainer^®^, Franklin Lakes, NJ, USA). The tubes were immediately centrifuged at 4 °C, the plasma transferred to 1.5 mL microfuge tubes, and stored in a −80 °C freezer until shipping on dry ice to Agilex Biolabs (Thebarton, Australia).

Plasma concentrations of R18D were determined using high-performance liquid chromatography tandem mass spectrometry (HPLC/MS; Agilex Biolabs). Samples were analyzed alongside calibrators prepared in a drug-free matrix and concentration in the test samples was determined from back calculation from the standard curve. Briefly, analytes were separated by HPLC on an XBridge Premier BEH Amide column, and the eluates were monitored by a Sciex 6500+ MS/MS detector in positive MRM mode (Sciex, Concord, ON, Canada). The extract was then assayed against a calibration curve. The data are acquired and integrated by the data acquisition system Analyst^®^ (Sciex) linked directly to the Sciex 6500+ MS/MS and then processed in Watson LIMS™ (Thermo Scientific, Waltham, MA, USA), where applicable. The lower and upper limits of quantification for R18D were 30 and 2400 ng/mL, respectively, as determined using 20 µL of rat plasma (note: mouse plasma was not available to use as a control), with an analytical run time of approximately 9 min per sample.

### 2.12. Statistical Analysis

Data was analyzed using ANOVA, followed by post hoc Fischer’s PLSD test (StatView; version 4.51, Abacus Concepts Inc, Berkeley, CA, USA). All data are presented as mean ± standard error of the mean (SEM). A *p* value < 0.05 was considered statistically significant for all analyses. Exact *p* values are reported for individual findings throughout the manuscript, where appropriate; values below 0.001 are reported as *p* < 0.001 or *p* < 0.0001. In vitro cell culture experiments were repeated independently at least two to three times, with a minimum of 3 to 6 biological replicates per assay. A minimum of four biological replicates were used for the Aβ_1–42_ aggregation biochemical assay.

## 3. Results

### 3.1. R18D Inhibits Aβ_1–42_ Aggregation in a Dose-Dependent Manner

**Figure 2:**
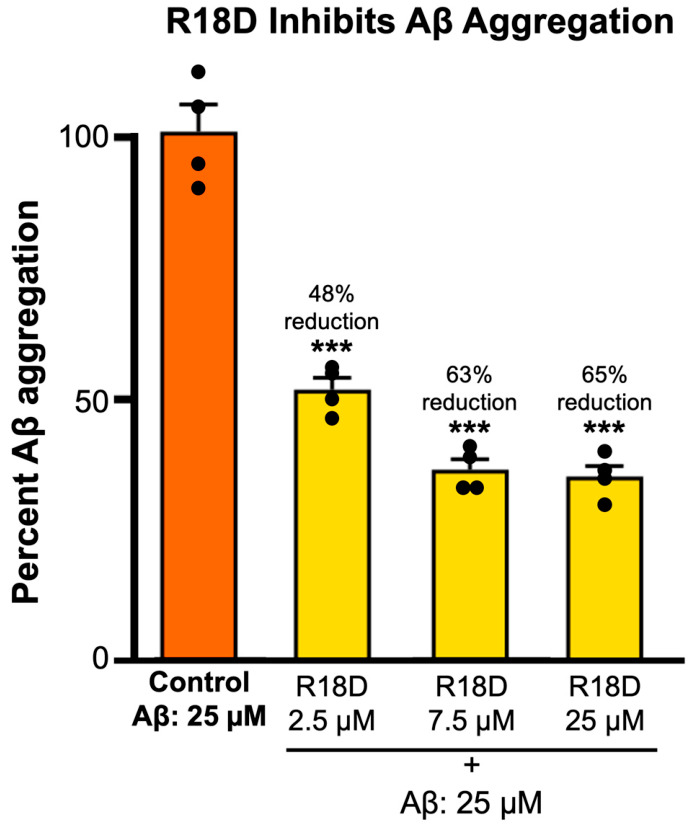
R18D reduces Aβ_1–42_ (Aβ) peptide aggregation in a cell-free assay. R18D (2.5, 7.5 and 25 μM) and Aβ (25 μM) incubated for 16 h at room temperature. ThT fluorescence values are from the 16 h endpoint (450 nm excitation/485 nm emission), taking the control fluorescence as 100% Aβ aggregation. Data are mean ± SEM; n = 4. *** *p* < 0.0001 when compared to the control. Control = vehicle plus Aβ.

In a cell-free assay, R18D markedly decreased the aggregation of monomeric Aβ_1–42_ in a concentration-dependent manner (Figure 2). R18D at 2.5, 7.5 and 25 µM reduced Aβ_1–42_ aggregation by 48%, 63% and 65%, respectively (*p* < 0.0001). These results confirm poly-arginine-18’s anti-peptide/protein aggregating properties [33,34] and highlight its potential to disrupt the pathological peptide/protein misfolding and aggregation, which is characteristic of neurodegenerative disorders such as AD.

### 3.2. Tetracycline Withdrawal Induces Intracellular Aβ-Induced Cytotoxicity in MC65 Cells

**Figure 3:**
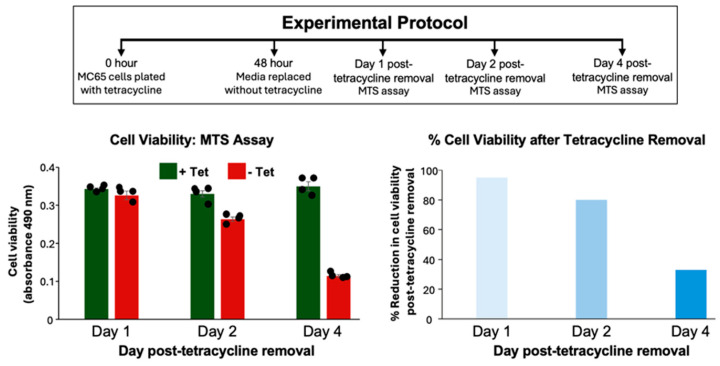
Processing of APP-C99 into Aβ_1–42_ peptide (Aβ) and Aβ toxicity in MC65 cells. Experimental design and Aβ toxicity in MC65 cells induced to overexpress APP-C99 for 4 days (96 h). MTS assay data represents absorbance measurements (490 nm) in control (non-overexpressing APP-C99 cells), and cells overexpressing APP-C99 and generating Aβ. Data are mean ± SEM; n = 4.

To confirm and characterize intracellular Aβ-induced cytotoxicity in MC65 cells, cultures were maintained in the presence or absence of tetracycline for 1 to 4 days, and cell viability was measured on days 1, 2 and 4 post-tetracycline removal. In the presence of tetracycline, MC65 cell viability remained stable from day 1 to day 4, whereas withdrawal of tetracycline resulted in a progressive decline in MC65 cell viability, with MTS absorbance decreasing by 5% at day 1, 20% at day 2 and 67% at day 4 (Figure 3). These findings demonstrate that tetracycline withdrawal induces a time-dependent increase in intracellular Aβ-associated cytotoxicity.

### 3.3. R18D Inhibits Intracellular Aβ Cytotoxicity in MC65 Cells

**Figure 4:**
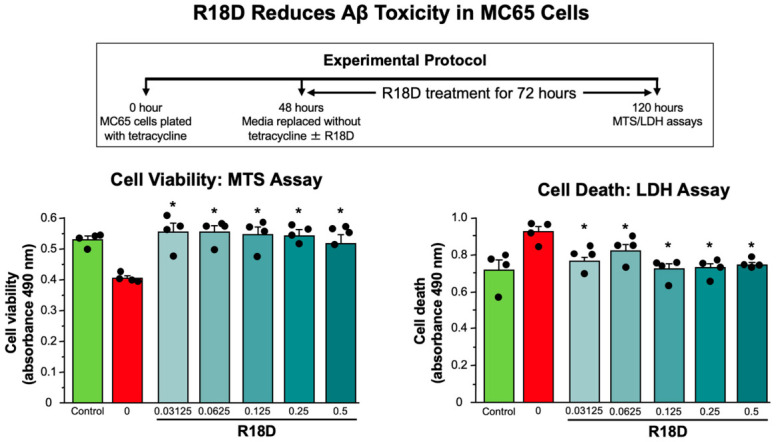
R18D reduces Aβ toxicity in MC65 cells overexpressing APP-C99. MC65 cells were induced to overexpress APP-C99 for 72 h in the presence of R18D. MTS and LDH assay data represents absorbance measurements (490 nm) in control (cells not overexpressing APP-C99), and cells overexpressing APP-C99 and generating Aβ with no treatment (0) or treatment with different concentrations of R18D (0.03125, 0.0625, 0.125, 0.25 and 0.5 µM). Data are mean ± SEM; n = 4. * *p* < 0.05 compared with no treatment (0).

To evaluate the efficacy of R18D in mitigating intracellular Aβ cytotoxicity, MC65 cells were cultured in the absence of tetracycline and simultaneously treated with R18D for 72 h (3 days), after which MTS and LDH assays were performed to assess cell viability and cell death, respectively. Based on the MTS assay, R18D maintained cell viability to control or above control levels at the 0.03125, 0.0625, 0.125, 0.25, and 0.5 µM concentration range (Figure 4). Similarly, based on the LDH assay, R18D resulted in reduced cell death at the 0.03125, 0.0625, 0.125, 0.25, and 0.5 µM concentrations to control or near control levels (Figure 4).

### 3.4. Tau Seeds Dose-Dependently Induce Intracellular Tau Aggregation in SH-SY5Y Cells and Cortical Neurons

To establish cell culture models of intracellular tau aggregation, SH-SY5Y cells and primary cortical neurons were exposed to increasing concentrations of tau seeds for 48 h.

**Figure 5:**
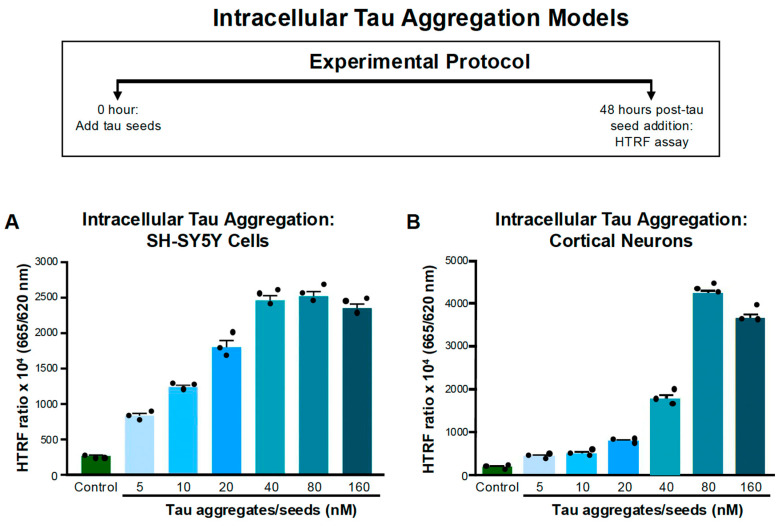
Tau aggregates/seeds dose-dependently induce intracellular tau aggregation in (**A**) SH-SY5Y cells and (**B**) primary cortical neurons. Cells were incubated with increasing concentrations of tau seeds (5–160 nM) for 2 h, after which the medium was replaced with seed-free media and cultures were maintained for a further 48 h. After 48 h, intracellular tau aggregates were quantified using a homogeneous time-resolved fluorescence (HTRF) assay. Fluorescence intensity was reported as the HTRF signal ratio (665 nm/620 nm × 10^4^), which provides a direct measure of tau aggregates. Data are mean ± SEM; n = 3.

Treatment of SH-SY5Y cells with tau seeds (0.01–0.16 µM) produced a dose-dependent increase in endogenous intracellular tau aggregation, which plateaued at between 0.04 and 0.08 µM before declining slightly at 0.16 µM (Figure 5A). Based on these results, subsequent experiments utilized the 0.08 µM tau seed concentration to induce intracellular tau aggregation in SH-SY5Y cells.

Similarly, treatment of primary cortical neurons with tau seeds (0.01–0.16 µM) dose-dependently increased endogenous intracellular tau aggregation (Figure 5B). Peak aggregation was achieved at the 0.08 µM tau seed concentration, with no further increase observed at 0.16 µM. Based on these results, subsequent experiments utilized the 0.08 µM tau seed concentration to induce intracellular tau aggregation in cortical neurons.

### 3.5. R18D Is Relatively Non-Toxic to SH-SY5Y Cells and Cortical Neurons

**Figure 6:**
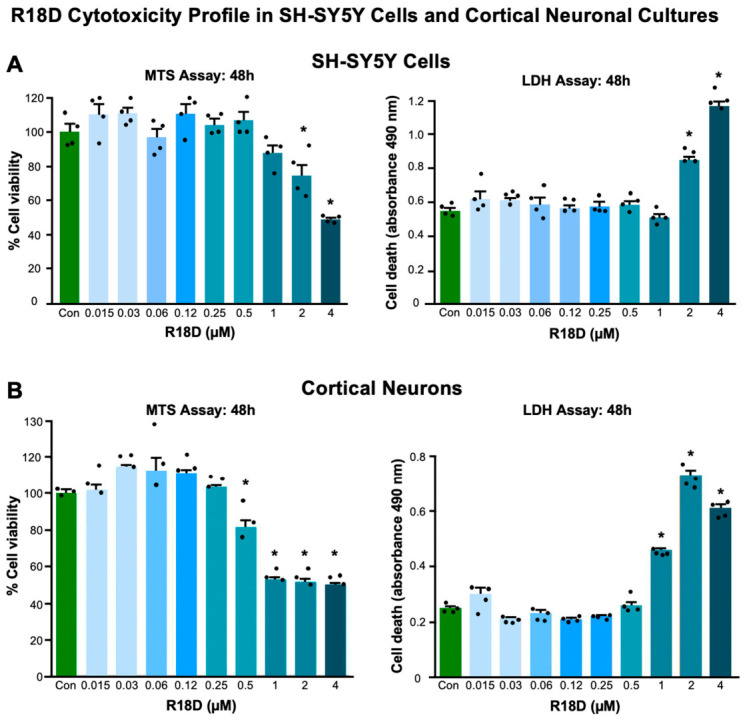
R18D cytotoxicity in (**A**) SH-SY5Y cells and (**B**) primary cortical neuronal cultures. Cells were incubated with increasing concentrations of R18D (0.015, 0.03, 0.06, 0.125, 0.25, 0.5, 1, 2 and 4 μM) for 48 h. Cell viability and cell death were assessed using an MTS and LDH assay, respectively. MTS absorbance measurements, reflecting metabolic activity, were normalized to represent cell viability, with the control (Con) set to 100% viability. LDH absorbance measurements at 490 nm reflect LDH release and cell death. Data are mean ± SEM; n = 3–4. * *p* < 0.05 when compared to the control (Con = untreated).

To establish an appropriate concentration of R18D for prolonged tau aggregation cellular studies, its cytotoxicity was evaluated in SH-SY5Y cells and cortical neurons after 24 and 48 h of exposure. Cell viability (MTS) and cell death (LDH) assays were used to examine R18D toxicity (Figure 6A).

In SH-SY5Y cells, MTS and LDH assays indicated minimal cytotoxicity of R18D at concentrations ≤ 1 μM (Figure 6A), whereas at ≥2 µM, significant toxicity was observed, which increased at the 2 and 4 μM concentrations. Interestingly, MTS measurements indicated a modest increase in metabolic activity at lower R18D concentrations (0.03125–0.25 µM) (Figure 6A). Based on the cytotoxicity data and to cover a broad concentration range, R18D at 0.0625, 0.125, 0.25, and 0.5 µM was used in subsequent SH-SY5Y cell tau aggregation studies.

In cortical neurons, the MTS assay showed that R18D at concentrations between 0.0015 and 0.25 μM had no to little effect on neuronal metabolic viability, whereas at 0.5 µM, a slight but significant decrease in metabolic activity was observed (*p* = 0.0373). Similarly, the LDH assays showed no cytotoxicity of R18D at ≤0.5 µM (Figure 6B). However, a modest but significant increase in cell death was observed at 1 µM (*p* < 0.001), which increased further at 2 and 4 µM. Consistent with observations in SH-SY5Y cells, MTS measurements indicated a modest increase in metabolic activity at lower R18D concentrations (0.03125–0.25 µM) (Figure 6B). Based on the toxicity data and to cover a broad concentration range, R18D at 0.0625, 0.125, 0.25 and 0.5 μM was used in subsequent neuronal cell tau aggregation studies, despite the potential for a modest toxic effect of the highest concentration (0.5 µM).

### 3.6. R18D Inhibits Intracellular Tau Aggregation

**Figure 7:**
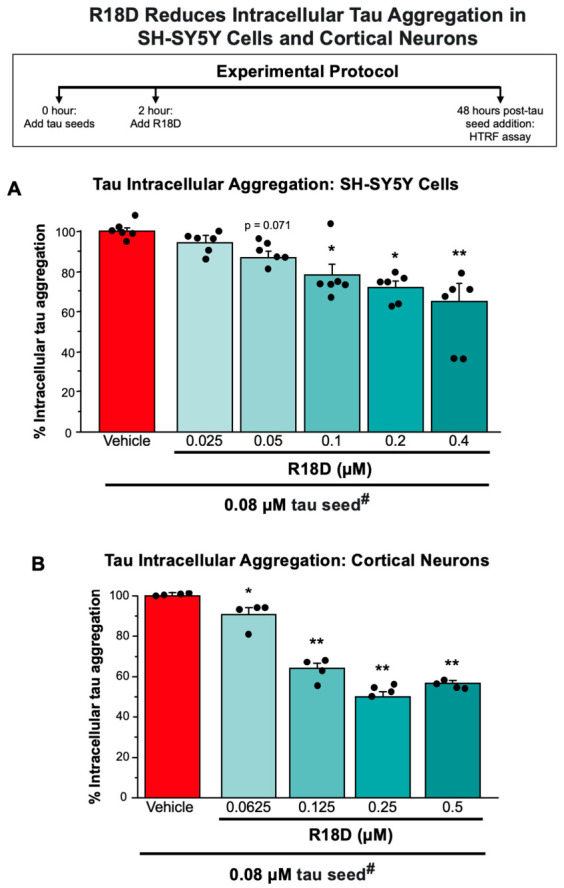
R18D inhibits intracellular tau aggregation in (**A**) SH-SY5Y cells and (**B**) primary cortical neurons. SH-SY5Y cells and cortical neurons were exposed to tau seeds (0.08 µM) for 2 h prior to treatment with R18D for 46 h. After the 48 h tau seed exposure, intracellular tau aggregation levels were measured using a homogenous time-resolved fluorescence (HTRF) tau aggregate assay. Fluorescence data has been transformed, with control (no tau aggregates and no R18D taken as background and subtracted from all experimental groups, and vehicle (tau seeds and no R18D) taken as 100% intracellular tau aggregation. Data are mean ± SEM; n = 4–6, * *p* < 0.05 and ** *p* < 0.0001 compared with vehicle. # Note: tau seed concentration is reduced by half (0.04 µM) following addition of R18D to SH-SY5Y cell and neuronal cell cultures.

To examine the ability of R18D to inhibit intracellular tau aggregation, SH-SY5Y cells and cortical neurons were exposed to tau seeds for 2 h to allow tau seed uptake, followed by treatment with R18D for 48 h (Figure 7A).

In SH-SY5Y cells, R18D produced a dose-dependent inhibition of intracellular tau aggregation (Figure 7A). R18D significantly reduced tau aggregation at 0.1, 0.2 and 0.4 µM by 21.7% (*p* = 0.004), 27.8% (*p* = 0.0004) and 34.8% (*p* < 0.0001), respectively. Similarly, in cortical neurons, R18D also dose-dependently inhibited intracellular tau aggregation, with inhibitory effects plateauing at 0.25 µM (Figure 7B). R18D significantly reduced tau aggregation at 0.0625, 0.125, 0.25 and 0.5 µM by 9.1% (*p* = 0.01), 36.1% (*p* < 0.0001), 49.9% (*p* < 0.0001) and 43.5% (*p* < 0.0001), respectively.

### 3.7. R18D Is Orally Bioavailable

**Figure 8:**
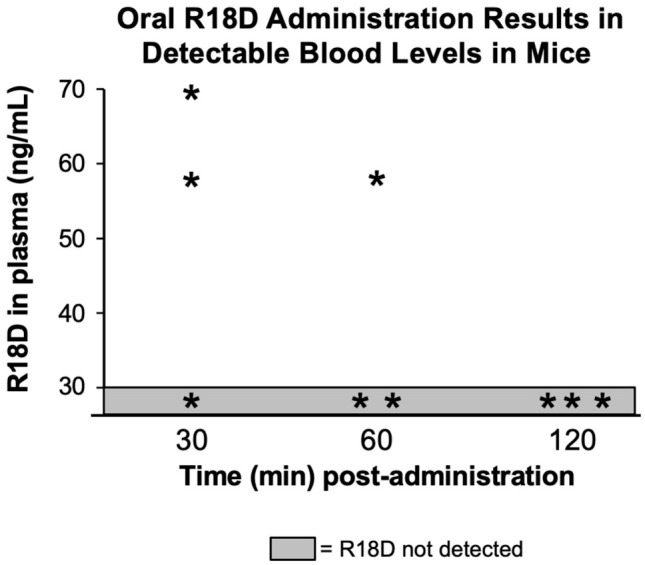
Detection and plasma concentration of R18D in mice following oral administration. R18D was administered via oral gavage at a dose of 20,000 nmol/kg. Three animals were analyzed for each time point post-R18D administration. Note: * represents the result for each individual mouse. Lower limit of detect is 30 ng/mL.

Following oral administration in mice, pharmacokinetic analysis detected R18D in three out of nine plasma samples. In two mice, R18D was detected at 30 min post-treatment at concentrations of 69.4 and 57.5 ng/mL, and in one mouse, at 60 min post-treatment at a concentration of 58.2 ng/mL (Figure 8).

## 4. Discussion

The accumulation of aggregated Aβ peptide and tau protein is a well-established pathogenic process in AD [35]; however, effective therapies aimed at minimizing these processes remain limited and new therapeutic approaches are urgently needed. In this pre-clinical study, using multiple in vitro models, we provide robust evidence that the novel poly-arginine peptide R18D attenuates key pathological processes associated with AD, including Aβ and tau aggregation as well as Aβ cytotoxicity. Together, these findings demonstrate the potential disease-modifying properties of R18D and highlight its promise as a therapeutic agent capable of targeting multiple mechanisms of AD pathology.

For example, R18D markedly inhibited Aβ aggregation in a cell-free assay and blocked the cytotoxicity associated with intracellular Aβ overexpression in human MC65 cells. Given that the accumulation of Aβ into neurotoxic amyloid plaques is the target of currently approved monoclonal antibody-based therapies, this is a significant finding. Importantly, even at a tenfold lower molar concentration (2.5 µM vs. 25 µM), R18D still reduced Aβ aggregation by approximately 50%. In 2021, we highlighted both the potential of cationic arginine-rich peptides (CARPs) to inhibit Aβ aggregation and the mechanisms likely underlying these effects [36]. Therefore, it was expected that the poly-arginine peptide R18D would effectively reduce Aβ aggregation. Moreover, we have recently reported that R18D can inhibit α-synuclein protein aggregation both in a cell-free assay and intracellularly within primary cortical neurons [34]. While the exact mechanisms whereby R18D and other CARPs inhibit Aβ aggregation have not been fully investigated, the positively charged guanidinium head group of arginine is thought to be a critical property, allowing interaction with aromatic (e.g., tryptophan) and negatively charged (e.g., glutamate) residues on proteins via cation-π interactions, hydrogen bonding and other steric electrostatic interactions [36]. These interactions are thought to stabilize partially unfolded proteins, inhibiting aggregation and maintaining protein solubility.

In addition to inhibiting Aβ aggregation, R18D was shown to inhibit intracellular Aβ cytotoxicity in human MC65 cells. Although Aβ is predominantly extracellular, it enters neurons and other cells via endocytic and non-endocytic pathways. Once inside, Aβ disrupts mitochondrial, endoplasmic reticulum and synaptic function, impairs the endosomal/lysosomal system, perturbs calcium homeostasis and destabilizes tau [37,38,39,40]. Because these intracellular effects predate amyloid plaque formation, they are likely to play a more critical role in neurotoxicity than extracellular Aβ dynamics, particularly in the early stages of AD. Consequently, these findings highlight the potential of R18D to counteract early pathogenic processes in the disorder and underscore the need to elucidate its precise protective mechanisms.

To examine the impact of R18D on tau aggregation, we successfully established two in vitro cell culture models of intracellular tau aggregation: one using the human neuroblastoma SH-SY5Y cell line, and the other using rat primary cortical neurons. Physiologically, tau is known to be involved in the assembly and stabilization of microtubules, as well as intracellular trafficking [41,42]. However, in neurodegenerative conditions such as in AD and other tauopathies (e.g., Pick’s disease, corticobasal degeneration, progressive supranuclear palsy and chronic traumatic encephalopathy), tau aggregation and accumulation play a key pathogenic role [43,44]. Furthermore, tau aggregates or seeds are thought to behave as prion-like proteins, leading to the spread of tau pathology through the CNS [43]. The tau in vitro models developed in this study appear to replicate the prion-like mechanisms underlying tauopathies, demonstrating that tau seeds can induce the intracellular aggregation of native tau protein in both SH-SY5Y cells and neurons. At low nanomolar concentrations, tau seeds induced a significant, dose-dependent increase in intracellular tau aggregation within a 48 h timeframe, which was markedly reduced by R18D. Importantly, R18D significantly reduced intracellular tau aggregation in both neurons and neuron-like cells, indicating that the effect is not cell-type-specific. This is particularly relevant as tau aggregation in tauopathies is not limited to neurons but also occurs in non-neuronal CNS cells, such as astrocytes [45] and oligodendrocytes [46].

It is likely that R18D inhibits tau aggregation through mechanisms similar to those described above for Aβ aggregation. Although the precise mechanisms whereby R18D inhibits the aggregation of AD-related peptides and proteins require further investigation, this study highlights its potential as a therapeutic agent capable of simultaneously targeting two major pathogenic processes in AD, in addition to its other potential cytoprotective properties [21,33,47]. Notably, the cytoprotective effects of R18D were confirmed in the intracellular Aβ-induced MC65 cell cytotoxicity model.

R18D has been used in multiple pre-clinical and clinical studies of acute brain injury (e.g., stroke, TBI and HIE) involving single-dose intravenous or intraperitoneal administration [22,27,28]. However, for chronic disorders such as AD, where long-term treatment would be necessary, oral administration is a more convenient route. In the proof-of-concept study presented here, we demonstrated that R18D, administered as a single oral dose, could result in detectable blood levels for up to 1 h post-treatment, thus supporting its potential as an orally active therapeutic. Given previous studies demonstrating the proteolytic stability [22,23] and cell-penetrating properties of R18D [48], as well as the reported oral stability of other D-enantiomer arginine-rich peptides [49,50,51,52], this result is expected. Due to variables such as the rate of absorption from the gastrointestinal tract and first-pass metabolism via the liver, the orally administered dose of R18D (20,000 nmol/kg) was considerably higher than doses typically delivered intravenously or intraperitoneally (e.g., 100 to 1000 nmol/kg), but consistent with the higher doses previously used in mice for D-enantiomer peptides via this route (e.g., RD2: 12,500 to 125,000 nmol/kg) [30,31,53]. While preliminary, these results highlight the oral bioavailability of R18D and pave the way for investigations to develop formulations that can further improve the peptide’s absorption from the gut. In parallel, pre-clinical and clinical investigations examining the safety as well as the efficacy of long-term repeated dosing of R18D will be required.

This study has several limitations. First, although R18D reduced Aβ aggregation, Aβ-induced cytotoxicity and intracellular tau aggregation, these effects were demonstrated in cell-free and in vitro cell assays, which may not fully recapitulate the in vivo environment. Furthermore, it remains to be determined whether the effective in vitro concentrations of R18D used in the present study can be achieved in vivo and translate into comparable therapeutic effects. Hence, it is essential to evaluate R18D in animal models that better reflect pathologic mechanisms associated with AD, particularly those involving both Aβ and tau. Second, the precise mechanisms by which R18D reduces Aβ and tau aggregation, as well as Aβ-induced cytotoxicity in MC65 cells, remain to be elucidated and require further investigation. In addition, further study is required to evaluate the effects of R18D on intracellular soluble and insoluble Aβ species in MC65 cells, and to characterize its cytoprotective dose–response relationship at lower concentrations in this model. Third, the intracellular tau model used in this study induced rapid tau aggregation over 48 h, but did not appear to cause any observable cytotoxicity; consequently, the model does not fully replicate the endogenous onset and progression of tau pathology that occurs in AD. This further emphasizes the need to examine the effects of R18D in animal models that are better suited to capture the temporal profile of tau-induced neurodegeneration. Fourth, the oral administration study examined only a single dose of R18D and did not assess other pharmacokinetic parameters, thereby limiting a comprehensive characterization of its pharmacokinetic profile. Therefore, additional oral dose–response studies with repeated dosing are required to fully evaluate the pharmacokinetic profile of R18D, confirm its brain penetration, and determine its suitability for chronic therapeutic use. Moreover, it remains to be determined whether blood concentrations of R18D achievable following oral administration are sufficient to reproduce the anti-amyloid and anti-tau effects observed in the present in vitro studies.

## 5. Conclusions

In conclusion, this study demonstrates that the poly-arginine peptide R18D effectively reduces Aβ aggregation in vitro, mitigates Aβ-induced cytotoxicity and inhibits intracellular tau aggregation. The ability of R18D to simultaneously target both Aβ and tau pathology, combined with its other potential multimodal neuroprotective mechanisms of action, makes it a unique and promising therapeutic agent for AD, with the potential to slow or halt disease progression. Crucially, the confirmed oral stability of R18D highlights its potential for development as an oral formulation suitable for long-term administration. Although these findings are preliminary, they strongly support the potential of R18D as a broad-acting neuroprotective agent for AD and other neurodegenerative disorders, warranting further evaluation in animal models and clinicl trials.

## Data Availability

The original contributions presented in this study are included in the article. Further inquiries can be directed to the corresponding author.
